# Analyses of inequalities in RMNCH: rising to the challenge of the SDGs

**DOI:** 10.1136/bmjgh-2018-001295

**Published:** 2019-06-24

**Authors:** Cesar Victora, Ties Boerma, Jennifer Requejo, Marilia Arndt Mesenburg, Gary Joseph, Janaína Calu Costa, Luis Paulo Vidaletti, Leonardo Zanini Ferreira, Ahmad Reza Hosseinpoor, Aluisio J D Barros

**Affiliations:** 1 Postgraduate Program in Epidemiology, Federal University of Pelotas, Pelotas, Brazil; 2 International Center for Equity in Health, Federal University of Pelotas, Pelotas, Brazil; 3 Department of Community Health Sciences, University of Manitoba, Winnipeg, Manitoba, Canada; 4 UNICEF USA, New York City, New York, USA; 5 Partnership for Maternal, Newborn & Child Health, World Health Organization, Geneva, Switzerland

**Keywords:** equity health, health systems evaluation, maternal health, child health, health services research

## Abstract

The Sustainable Development Goal (SDG) 17.18 recommends efforts to increase the availability of data disaggregated by income, gender, age, race, ethnicity, migratory status, disability and geographic location in developing countries. Surveys will continue to be the leading data source for disaggregated data for most dimensions of inequality. We discuss potential advances in the disaggregation of data from national surveys, with a focus on the coverage of reproductive, maternal, newborn and child health indicators (RMNCH). Even though the Millennium Development Goals were focused on national-level progress, monitoring initiatives such as Countdown to 2015 reported on progress in RMNCH coverage according to wealth quintiles, sex of the child, women’s education and age, urban/rural residence and subnational geographic regions. We describe how the granularity of equity analyses may be increased by including additional stratification variables such as wealth deciles, estimated absolute income, ethnicity, migratory status and disability. We also provide examples of analyses of intersectionality between wealth and urban/rural residence (also known as double stratification), sex of the child and age of the woman. Based on these examples, we describe the advantages and limitations of stratified analyses of survey data, including sample size issues and lack of information on the necessary variables in some surveys. We conclude by recommending that, whenever possible, stratified analyses should go beyond the traditional breakdowns by wealth quintiles, sex and residence, to also incorporate the wider dimensions of inequality. Greater granularity of equity analyses will contribute to identify subgroups of women and children who are being left behind and monitor the impact of efforts to reduce inequalities in order to achieve the health SDGs.

**Summary box**

- The Sustainable Development Goals (SDGs) call for better and more finely grained analyses on health equity, presenting a challenge to researchers and designers of health surveys.
- With currently available data, it is possible to further equity analyses, and we present innovative ways to deal with equity analysis to incorporate absolute wealth, ethnicity and intersectionality between different dimensions of inequalities.
- The analytical examples provided here show how new approaches may lead to deeper understanding of patterns of inequality and thus help health managers and policy makers fine-tune their policies and programmes.
- The challenges presented here will need to be tackled by survey designers and implementers, data analysts and all those involved in capacity strengthening towards the achievement of SDG 17.18.

## Background

The Millennium Development Goals (MDGs, 1990–2015 failed to address within-country health inequalities (http://www.un.org/millenniumgoals/), with country performance being solely assessed according to national level progress on health goals (MDGs 4, 5 and 6).^1^ Nevertheless, the literature on socioeconomic inequalities in health increased rapidly during the MDG era^2^ and from 2005 the Countdown to 2015 initiative provided regular reports on within-country health inequalities according to wealth, education, gender and place of residence.^3^ Starting with the World Bank,^4^ equity analyses have permeated the documents and websites of international organisations. The WHO’s Health Equity Monitor (http://www.who.int/gho/health_equity/en/) and its publication on the State of Inequality,^5^ and analyses by Unicef^6 7^ have contributed to mainstreaming equity considerations. In many developing countries, however, the extent to which disaggregated analyses are presented and used for policies and programmes is still quite limited.

The launch of the Sustainable Development Goals (SDGs) in 2015 more clearly brought equity to the forefront (https://sustainabledevelopment.un.org). The third SDG (‘ensure healthy lives and promote well-being for all at all ages’) has an intrinsic equity component, and the tenth goal (‘reduce inequality within and among countries’), although focused on economic inequality, also highlights the importance of reducing disparities. SDG 17.18 on data, monitoring and accountability specifically addresses the need to increase ‘the availability of high-quality, timely and reliable data disaggregated by income, gender, age, race, ethnicity, migratory status, disability, geographic location and other characteristics relevant in national contexts’.

In this paper, we address the challenges presented by SDG 17.18, with examples from coverage of reproductive, maternal, newborn and child health (RMNCH) interventions in low-income and middle-income countries (LMICs). We focus on data from national surveys, which provide the most comprehensive and comparable assessments of intervention coverage, while noting that administrative data from health information systems are also useful for demonstrating some dimensions of inequalities, particularly geographic. We report on the experience of the Countdown to 2030 Equity Technical Working Group, based at the Federal University of Pelotas, Brazil, in exploring new ways of analysing and presenting health inequalities according to a wide range of dimensions.

## Data sources

Data for the present analyses were retrieved from publicly available nationally representative health surveys carried out from 1991 to 2016, including the Demographic and Health Surveys (DHS), Multiple Indicator Cluster Surveys (MICS) and Reproductive Health Surveys (RHS). Our analyses relied on data from up to 349 surveys carried out in 113 countries (www.equidade.org/surveys), henceforth referred to as the Pelotas database. All surveys used multistage cluster sampling designs to obtain nationally representative data. Standardised questionnaires were used to collect information from women of reproductive age living in the sampled households. Ethical approval was the responsibility of the institutions in charge of each survey. More details on DHS, MICS and RHS are available elsewhere.^8 9^

Most examples provided below refer to inequalities in coverage with institutional delivery, that is, the proportion of births that take place in a hospital or other type of health facility. For the intersectionality of child sex and wealth, we report on full immunisation coverage, or the proportion of children aged 12–23 months who have received at least three doses of diphtheria-pertussis-tetanus, three doses of polio, one dose of measles and one dose of BCG vaccines. These interventions were selected to cover the two most common delivery channels, health facility (institutional delivery) and community (vaccination). In addition, institutional delivery is the most inequitably distributed intervention in most countries, whereas immunisation coverage tends to be more equitable. Further information on the indicators is available at http://www.equidade.org/resources/indicators.pdf).

## PANEL 1. Sample sizes for disaggregated analyses

RMNCH survey sample sizes have increased over time due to the growing interest in disaggregated analyses. The median number of children aged less than 5 years in the surveys included in the Pelotas database (DHS, MICS and RHS) increased from 4523 in the 1991–1999 period to 5140 after 2010 (The mean sample size increased from 4827 in 1990-99 to 7321 in 2010 or later, an increase that was driven by some large recent surveys).

Table 1 shows the median denominators for key RMNCH coverage indicators, by wealth quintiles. The median provides a more realistic picture of the sample sizes available for analyses than the means, which are distorted by a few very large surveys, such as the recently released 2015 India DHS. The median numbers of households and of women are similar in all quintiles, but due to higher birth rates the number of under-five children in the poorest quintile is almost twice as large as in the richest quintile. Even larger imbalances are observed for case-management indicators, given the much higher incidence of diarrhoea and suspected pneumonia among the poor. Indicators of exclusive breastfeeding and immunisations, which are based on restricted age ranges, also have relatively small denominators.

**Table 1:** Sample sizes for selected domains of RMNCH indicators in 129 surveys from 2010 to 2016

Frequently used denominators	Examples of indicators based on each denominator	Median sample size by wealth quintiles				
		Poorest	Second	Third	Fourth	Richest
All households with women aged 15–49 years or children under 5 years	Water, sanitation, hygiene	2507	2352	2188	2215	2241
Sexually active* 15–49-year-old women and girls	Contraceptive coverage	1683	1626	1605	1570	1526
Live births in the past 2–3 years to 15–49-year-old women and girls†	Antenatal, delivery and postnatal care	1001	885	826	697	481
Children aged <2 years	Early initiation of breastfeeding	765	644	578	504	418
Children aged <6 months	Exclusive breastfeeding	183	157	136	121	103
Children aged 12–23 months‡	Immunisations	376	319	299	262	224
Children aged <5 years with diarrhoea in the past 2 weeks	Oral rehydration therapy or solution	303	232	200	158	99
Children aged <5 years with suspected pneumonia in the past 2 weeks	Care-seeking for pneumonia	109	86	63	52	45
Children aged <5 years	Bednets, anthropometric indicators	1334	1139	1033	950	728

*Some surveys only ask this question for women who are married or in union.

†Denominators refer to women who delivered a live child in the past 2 years (MICS) or 3 years (DHS). Postnatal care refers to women giving birth in the past 2 years for both types of surveys.

‡In some countries, the denominator includes children aged 15–26 or 18–29 months, to take into account the immunisation calendars.

DHS, Demographic and Health Surveys; MICS, Multiple Indicator Cluster Surveys; RMNCH, reproductive, maternal, newborn and child health indicators.

## Income, wealth and socioeconomic position

Potential indicators for assessing socioeconomic position through surveys in LMICs include household assets, consumption expenditure, income, education, occupation and subjective measures such as participatory wealth rankings and self-rating.^10^ Each approach has strengths and limitations. Education of the woman or mother and occupation of the father were the most frequently used stratifiers in the RMNCH literature up to the 1990s,^11^ but these are not mentioned in SDG 17.18. Consumption and income are difficult to measure in LMICs and require specialised living conditions surveys. A major breakthrough took place in the late 1990s, when wealth indices derived from household assets, construction materials and access to utilities were introduced.^12^ These indices were first incorporated by DHS,^13^ and later by MICS, and rapidly became a standard tool in survey-based equity analyses.^1 4^

Surveys regularly collect information on household goods (eg, refrigerators, beds), characteristics of the dwelling (materials used for the walls, floor and roof, water source, sanitation) and access to utilities (electricity, internet). Other variables related to economic position such as house and land ownership, and assets relevant to rural areas like livestock are also recorded. To estimate an asset-based wealth index, these variables are included in a principal component analysis (PCA) for all households in the sample, excluding variables that are only relevant urban or rural areas. Next, two separate PCAs are carried out for urban and rural households, including all relevant variables in each domain. Using the first component or each PCA, the separate urban and rural scores are used to predict the joint PCA scores through linear regression. The predicted values of this regression is used as an adjusted combined score for all households, usually used to split the sample into equal sized groups, most often quintiles.^14^

Asset indices present limitations,^10 15^ notably their sensitivity to different choices of assets,^16 17^ the possibility of confounding by urban or rural residence (as poor households prevail in rural areas)^18 19^ and their inability to discriminate among the very poor due to lack of variability in assets. A further limitation is that asset indices assess relative, rather than absolute socioeconomic position.^20 21^ The poorest quintile in one country may be richer in absolute terms than the richest quintile in another country.

The advantages of using an asset index include the ease of calculation, the use of standard approaches across countries, the fact that each quintile, by definition, represents a similar proportion of all households, and the consistent associations with more complex measures of socioeconomic position.^18^ The strong associations between asset indices and most RMNCH indicators is a compelling demonstration of their usefulness for documenting inequalities.^4 22^

The large majority of current analyses of socioeconomic inequalities in LMICs use asset index-based wealth quintiles. Next, we present analytical approaches that go beyond quintiles.

## Wealth deciles

**Figure 1:**
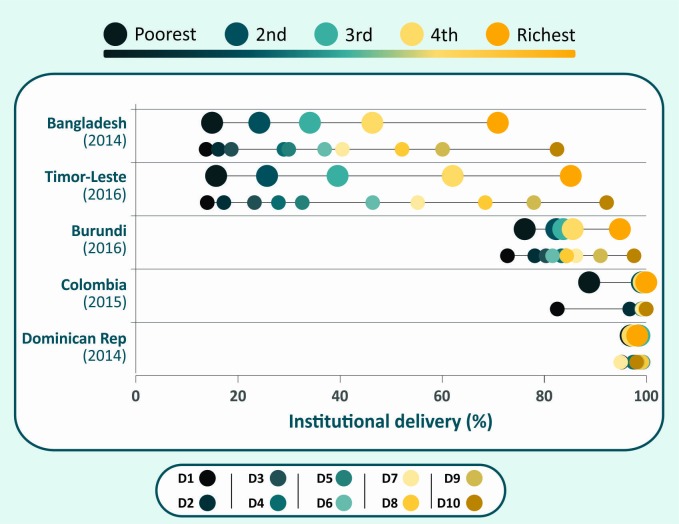
Institutional delivery coverage according to wealth quintiles and deciles in selected countries.

Over time, the increasing average size of surveys over time (Panel) allows finer disaggregation of health outcomes than was possible in the past. For example, wealth deciles may be used instead of quintiles.^23^ Figure 1 shows equiplots (www.equidade.org/equiplot) of institutional delivery coverage for five countries, by quintile and by decile. In Bangladesh, Timor Leste and Burundi, the richest decile has substantially higher coverage than all other deciles. These countries show ‘top inequality’ patterns, that is, inequalities are driven by the wealthiest.^24^ In contrast, in countries such as Colombia where there is ‘bottom inequality’, coverage is markedly lower in the first decile in comparison to all the others. In Dominican Republic, coverage is very high and inequality small, and the use of deciles does not add much to the analyses by quintile

Disaggregation of coverage data by deciles rather than quintiles often reveals further within-country inequalities. An analysis of coverage with skilled attendant at birth in 46 surveys showed that the average difference in coverage between the richest and poorest deciles was 52.7% points, compared with 45.0% points for the difference between the extreme quintiles.^23^ A limitation of the use of deciles is sample size, particularly for indicators such as careseeking for childhood illnesses which are less common among children from rich families (table 1). Use of deciles may contribute to advocacy efforts, monitoring inequalities over time and targeting health interventions.

### Absolute income

Although SDG 17.18 specifically mentions the need to disaggregate indicators by income, few RMNCH surveys collect such information for reasons discussed above.^25^ This limitation has motivated researchers to develop methods for estimating absolute wealth or income, to allow comparisons across countries and over time. The simplest approach—the ‘absolute wealth index’—is an arithmetic sum of common assets measured in several country surveys,^26 27^ under the implicit assumption that the sum of assets has the same value in different societies. More sophisticated approaches are the ‘international wealth index’^28^ and the ‘comparative wealth index’.^29^

Ascribing a dollar value to wealth quintiles requires information on national income levels (eg, per capita gross national product) and income distribution, which are available from economic surveys. Hruschka and Brewis used the national income share by quintile to estimate the absolute income, which was then applied to survey-derived wealth quintiles.^30^ A limitation of this approach is that information on income share is available for relatively few countries. Harttgen and Vollmer used a similar approach, but instead of income share relied on the Gini index for income distribution which is available for virtually every country.^31^ The assumption behind both approaches is that asset-index scores are a good approximation for household income ranking. Using the Harttgen and Volmer approach, Fink *et al* 20 showed that absolute incomes are markedly superior to relative wealth quintiles in terms of predicting stunting prevalence in under-five children across and within countries over time.

Analyses of health intervention coverage by absolute income are useful for assessing country performance. Countries with higher coverage than others at similar levels of income are likely to have benefited from effective policies and programmes. Analyses of time trends are also useful for evaluating whether progress in a country can be attributed solely to economic growth or whether interventions in the health or other sectors played a role.

**Figure 2:**
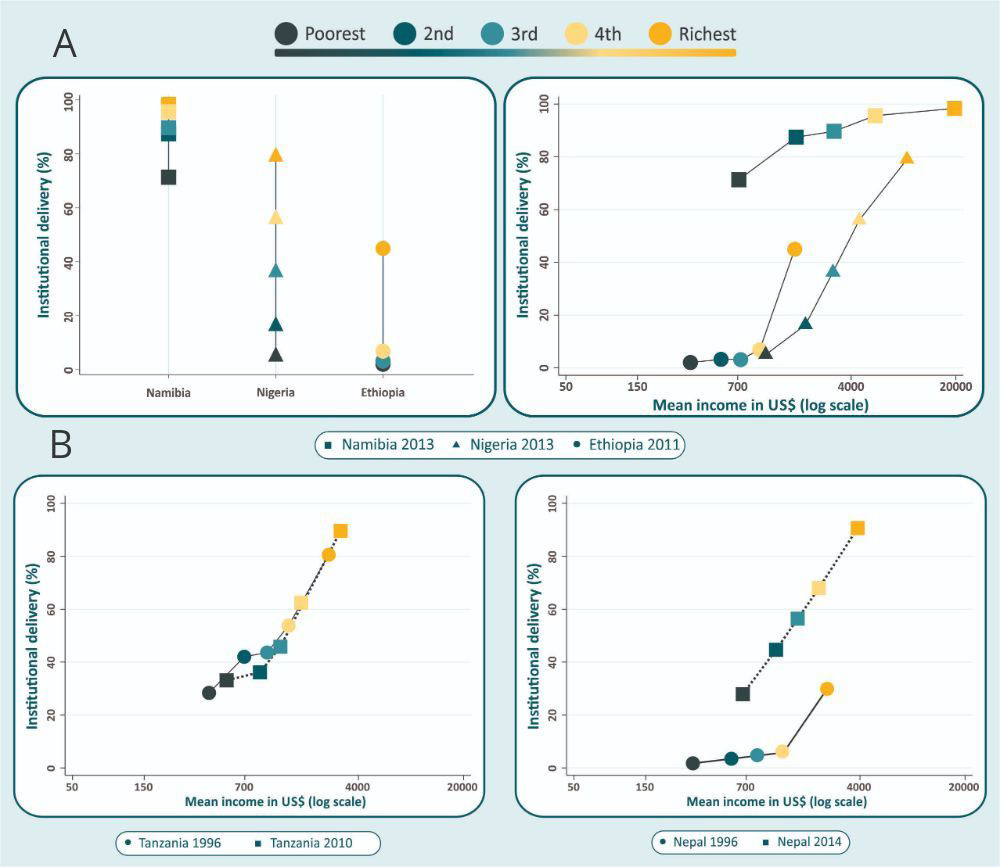
Institutional delivery coverage according to (A) wealth quintiles (left-hand panel) and to absolute income (right-hand panel) in selected countries and (B) at two points in time in Tanzania (left-hand panel) and Nepal (right-hand panel).

**Figure 3:**
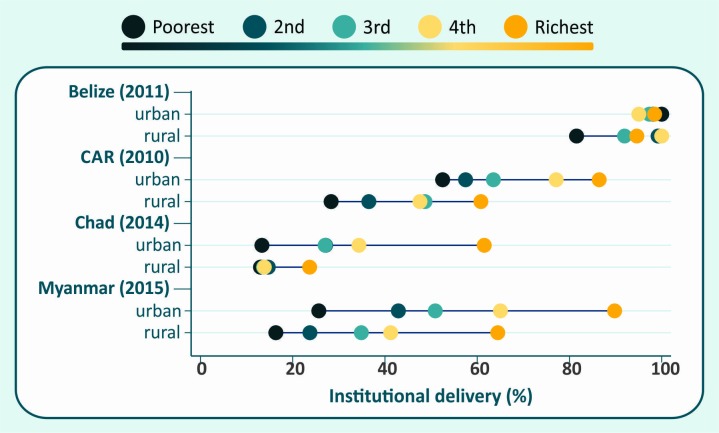
Institutional delivery coverage according to wealth quintiles and place of residence in selected countries.

We illustrate the advantages of absolute incomes relative to wealth quintiles in figures 2 and 3. The left-hand panel in figure 2A vertical equiplot of institutional delivery coverage shows that for every quintile of relative wealth, coverage in Namibia is higher than in Nigeria and coverage in Ethiopia is lowest. The right-hand panel—in which coverage is plotted against the mean absolute income in each quintile—shows that Ethiopia is considerably poorer than Nigeria and Namibia. These two countries show roughly similar levels of absolute income for each quintile, but coverage in Namibia is markedly higher than in Nigeria for the same income level, particularly in the poorest quintiles. Even in Ethiopia, where overall coverage is lower, the top quintile has approximately the same income as the second quintile in Nigeria, with much higher coverage.

Figure 2B shows changes in absolute income and institutional delivery coverage over time. In Tanzania, absolute income and coverage increased very slightly between 1996 and 2010. The small increases in coverage in each quintile can be explained by the similarly small increase in absolute income. In contrast, the data from Nepal show important increase in coverage for every quintile between 1996 and 2014, which cannot be attributed to changes in income, which also increased at a lower rate. Similar results were found for skilled attendant at birth.^32 33^

## Ethnicity

Among the several dimensions of inequality listed in SDG 17.18, ethnicity is particularly difficult to measure in a comparable way across countries.^32^ It is not surprising, therefore, that few multicountry studies of ethnic inequalities are available, compared with the large number of analyses focusing on inequalities associated with wealth, education, age or place of residence. In addition, most published studies on ethnic inequalities have focused on mortality, fertility and nutrition outcomes,^32 34^ rather than on RMNCH coverage.

Proxies for ethnicity in RMNCH surveys include language and self-reported ethnic affiliation or skin colour. We reviewed 129 DHS and MICS carried out since 2010 and found that 74% provide some information on ethnicity. The type of information varies by region. In Africa, surveys typically ask about ethnic group affiliation, with the number of categories ranging from 11 (Congo, Cameroon) to over 300 (Nigeria), with five countries listing 30 or more ethnic groups. In Eastern Europe, similar questions are asked but fewer than 10 categories are recorded. In Asian surveys, the number of ethnic groups range from two (Bangladesh and Thailand) to 105 (Nepal). For some countries, the ethnic categories recorded vary markedly from one survey to another.

The population in Latin American and the Caribbean (LAC) derives from three major migratory currents, which makes analyses of broad ethnic groups simpler than for other regions. Surveys usually ask about ethnic group affiliation, skin colour or language spoken at home. Such information may be used to identify three groups: indigenous (involving many distinct nations), afrodescendants and the reference group. The latter includes subjects who either did not declare themselves as indigenous or afrodescendants, do not speak an indigenous language at home or who considered themselves white.^35^ Because marriage between different ethnic groups is common in the region, this reference category includes a substantial proportion of individuals with mixed European, indigenous and African ancestries. Figure 3 shows coverage by institutional delivery for 11 countries in LAC. In spite of the limitations of the classification, coverage with institutional delivery was markedly lower among indigenous women in most countries, except for Uruguay. Afrodescendants tended to show similar levels to those in the reference group.

Even in LAC, where the classification of ethnicity may be simpler, there are challenges. Questionnaires often included many categories, requiring local expert advice for recoding. The proportion of each group in the surveys was often different from national censuses, possibly because of different questions or sampling strategies. In several countries, the number of afrodescendants or indigenous women was rather small, resulting in poor precision. Issues related to discrimination and persecution may also affect self-classification in surveys^36^ or even preclude survey designers from asking the question.

Robust analyses of ethnic disparities will require improved design of questionnaires and sampling strategies, to ensure that such information is collected in a systematic way and that ethnic minorities are adequately represented in the sample. Greater involvement of different ethnic groups in the design and analysis is also essential.

## Intersectionality

The study of intersectionality, or the interaction of different social stratifiers, is receiving increased attention.^37^ It requires stratification of results by two or more equity dimensions, for example, double or triple disaggregation. Sample sizes allowing survey data are well suited for such analyses.

Although many studies address coverage with delivery care interventions according to wealth or place of residence,^38–41^ few have investigated the intersectionality between these two stratifiers. In most countries, the poor are concentrated in rural areas. Yet, neither rural nor urban populations are homogeneous. The urban poor may show coverage levels well below the rest of the urban population and possibly lower than the rural population.^42^ Double disaggregation by urban/rural residence and wealth quintiles allows the examination of RMNCH outcomes in 10 categories of households. This allows comparison of outcomes among urban and rural residents with similar wealth levels, although sample sizes differ in the 10 cells, typically with the smallest groups being urban poor and rural rich.

Analyses by Matthews *et al* 42–44 showed that the urban poor did not necessarily have better access to services than the rural poor, despite being closer to them.^42^ They proposed a common pathway to describe progress to universal health coverage, with the urban rich being the first to obtain universal coverage followed by the rural rich, the urban poor and lastly the rural poor.^43^

**Figure 4:**
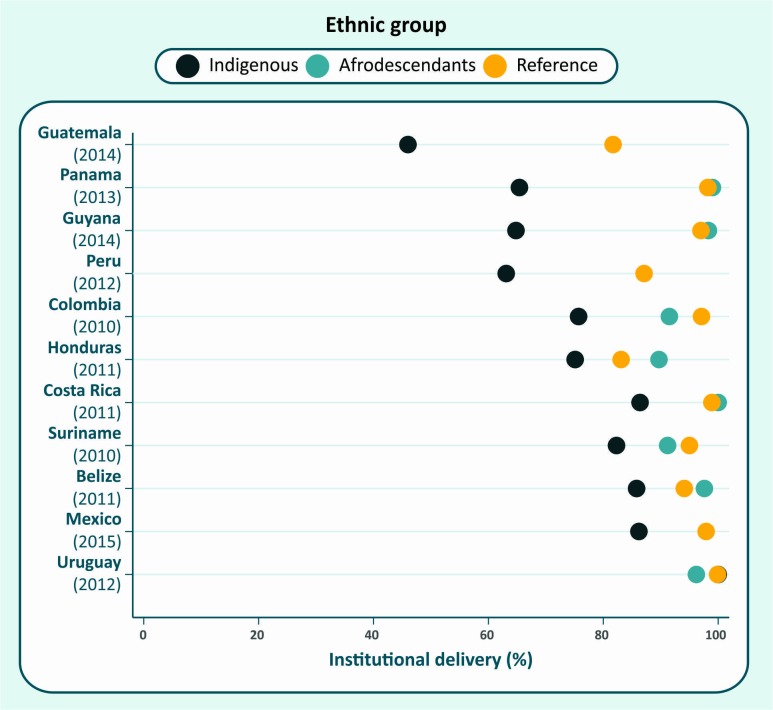
Institutional delivery coverage in indigenous, afrodescendant and reference group women in selected countries in Latin America and the Caribbean.

Our analyses of institutional delivery coverage in countries with surveys since 2005 illustrate the limitation by sample size, with only 45 of 103 countries having 25 or more women in all 10 cells. Nevertheless, the analyses showed markedly variable patterns by country. In figure 4, we show the results for four selected countries. In Belize, only the poorest rural women were being left behind. In the Central African Republic (CAR), every rural quintile is behind its urban counterpart, with the richest rural quintile presenting lower coverage than the middle urban quintile. In Chad, there was marked inequality among the urban quintiles, but in rural areas coverage was very low and inequalities small. In Myanmar, both urban and rural areas showed marked inequalities, with rural women lagging behind urban women within each quintile.

Among the 45 countries analysed, only Malawi and Thailand showed significantly lower coverage in the poorest urban quintile than in the poorest rural quintile. These results challenge the notion that the urban poor would be the most disadvantaged. This is consistent with Fink *et al*, who identified urban slum areas in 73 LMICs, and showed that children from slums had significantly better health indicators than those from rural areas, but were worse-off than richer urban children.^45^

Analyses of the intersectionality between wealth and residence, wealth and sex, or any other two or three stratifiers, may advance the understanding of the nature and degree of inequalities, compared with simpler analyses using a single stratification variable. The study of intersectionality may point out to groups that are being left behind in each country and highlight the need for tailored policy interventions.

## Other dimensions of inequality

SDG goal 17.18 calls for other dimensions of inequality, such as age, migratory status, disability and geographic location. Some of these dimensions are quite difficult to measure and some are just starting to be included in national health surveys. Only a few surveys have asked about migration so far. And disability is another very little explored area, with few exceptions. Geographic location is regularly recorded in surveys, but usually in large subnational areas such as regions or provinces. Despite more recent surveys including geotagging of sample clusters,^46^ the sample is not designed to allow for very fine geographical stratification and the analyses that have been done so far rely heavily on modelling in order to produce estimates for grids commonly sized at 5 by 5 km. Given space limitations for this article, we present a more detailed discussion on additional dimensions of inequalities in the webannex (online supplementary file).

10.1136/bmjgh-2018-001295.supp1 Supplementary data

## Conclusion

The SDGs have raised the bar for analyses of inequalities in health. We described the main challenges that are posed, with a focus on the analyses of survey data on RMNCH coverage indicators. We also presented some of the benefits of newer analytical approaches, including finer stratification by wealth using deciles, the use of predicted incomes for asset-based quintiles and analyses of intersectionality. While most of these analyses are straightforward using existing survey data, they may be affected by small sample sizes, particularly for outcomes such as vaccine coverage or management of childhood illnesses, which are based on subsamples of children covered by the survey, or analyses of outcomes for young adolescents.

Other types of stratification variables are proving more difficult to tackle. Data on proxies for ethnicity are now collected in three out of every four surveys, but the numbers of groups are large in many surveys, again leading to issues with sample sizes. Pooling different ethnic groups for analysis purposes is not trivial, and decisions on recoding may be challenged by specific ethnicities. Data on migration, displaced populations and disability are simply not available in most RMNCH surveys.

The examples of analyses presented above were restricted to indicators of intervention coverage. The same approaches apply the study of inequalities in other indicators such as undernutrition or overweight, water and sanitation and child mortality. Regarding the latter, it is important to consider sample size limitations that affect the relatively rare event of death; such limitations may result in imprecise estimates when the sample is stratified into a large number of subgroups.

Although the examples in this article refer to multipurpose, standardised national RMNCH surveys, many other data sources may be subjected to similar analyses to identify and monitor progress for groups that are being left behind. Disease-specific surveys such as Malaria Indicator Surveys (http://www.malariasurveys.org/) and AIDS Indicator Surveys (https://dhsprogram.com/what-we-do/survey-types/ais.cfm), for example, include questions that allow calculation of the asset index through principal component analyses, and many reports provide breakdown of outcomes by wealth quintiles. In some instances, special surveys are needed to capture information among marginalised populations that are not easily captured in general population surveys. Use of health facility and other administrative data for equity analyses is usually restricted to tabulations by sex, age and residence. Routinely reported health facility data provide continuous information which provides an opportunity for finer geographical breakdowns than is usually the case for surveys. There are, however, major challenges related to the quality of data, the estimation of the target populations by district, the lack of information on—for example—wealth or ethnicity, and on how to handle multiple contacts with a facility by an individual.

Equity analyses at country and global level are essential for documenting which interventions and programmes are more (or less) equitable, and whether there has been progress over time. However, the most important use of equity analyses takes place at country level, where results can be fed back to policymakers and programme managers. Disaggregated analyses can show which population subgroups are being left behind, leading to an understanding of the bottlenecks that must be overcome in order to achieve equitable progress.^47^ Equity analyses may require increasing the sample in routine surveys. Combining survey data with administrative information may allow greater granularity and help target programmatic efforts.^48^ Last, advocacy materials can use results of equity analyses to promote human rights and achieve universal health coverage, for example by focusing on mothers and children who are failing to receive any of a set of well-established preventive interventions.^49^

SDG goal 17.18 is much welcome, but it has upped the ante for equity analyses. It calls for strengthening country capacity by 2020 in the production and use of disaggregated statistics. This will require concerted efforts by national governments, bilateral and international organisations, and by multi-stakeholder initiatives such as the Countdown to 2030 (www.countdown2030.org). The 2020 deadline for building country capacity in equity analyses is ambitious. We must start right away.
