# Supplementary material for: Analyses of inequalities in RMNCH: rising to the challenge of the SDGs
Source: BMJ Glob Health. 2019 Jun 24;4(Suppl 4):e001295. doi: 10.1136/bmjgh-2018-001295 (PMC6590961; doi:10.1136/bmjgh-2018-001295)
Supplement: Supplementary data [file bmjgh-2018-001295supp001.pdf]

WEBAPPENDIX

We present here a more detailed discussion on additional dimensions of inequality: migratory status, geographic location, age, disability and education.

Migratory status

SDG 17.18 also calls for disaggregation of data by migratory status. The amount of information available in RMNCH surveys is limited and when available, unstandardized. Of 129 surveys carried out since 2010, 39 presented information on migration or proxy variables such as language or nationality that may be used to identify migrants. Among the 39 surveys, only six (Bosnia and Herzegovina, Colombia, Kenya, Nepal, Myanmar and Pakistan) provided detailed information on migration or internal displacement, including the reason for moving, with an option being war or conflict; 21 had information on nationality, 16 of which only had two options (national or foreigner); eight had information on internal migration from rural to urban areas; and four provided information on time at the present address and region of previous residence. For the six countries with information on the reasons for displacement, those who migrated due to violence or conflict may be compared with the rest of the population, if sample sizes allow.

Lack of comparability of available information suggests that multi-country analyses are not possible; analyses will have to be carried out on a country by country, opportunistic basis. For example, surveys carried out in neighboring countries at around the same point in time may allow comparing migrants to women and children living in their countries of origin as well as in their destination countries. Figure A1 is based on the 2012 DHS in Haiti and the 2014 MICS in the Dominican Republic. For all interventions other than oral rehydration and immunization, Haitian migrants living in the Dominican Republic had higher coverage than Haitians living in Haiti, but generally not as high as native Dominicans.

Geography

The analyses of geographic inequalities within a country are particularly useful for helping target interventions. Ideally, surveys should have large sample sizes, to provide representative results for small geographic areas. The median number of subnational regions in surveys carried out since 2010 was nine, and 32 surveys had six or fewer regions. Only a handful of countries, including Afghanistan, India, Indonesia, Malawi, Nigeria, Peru and Tanzania provided representative results for 20 or more regions. For the vast majority of countries, therefore, the number of geographic regions represented in the surveys are insufficient for precise geographic targeting of interventions.

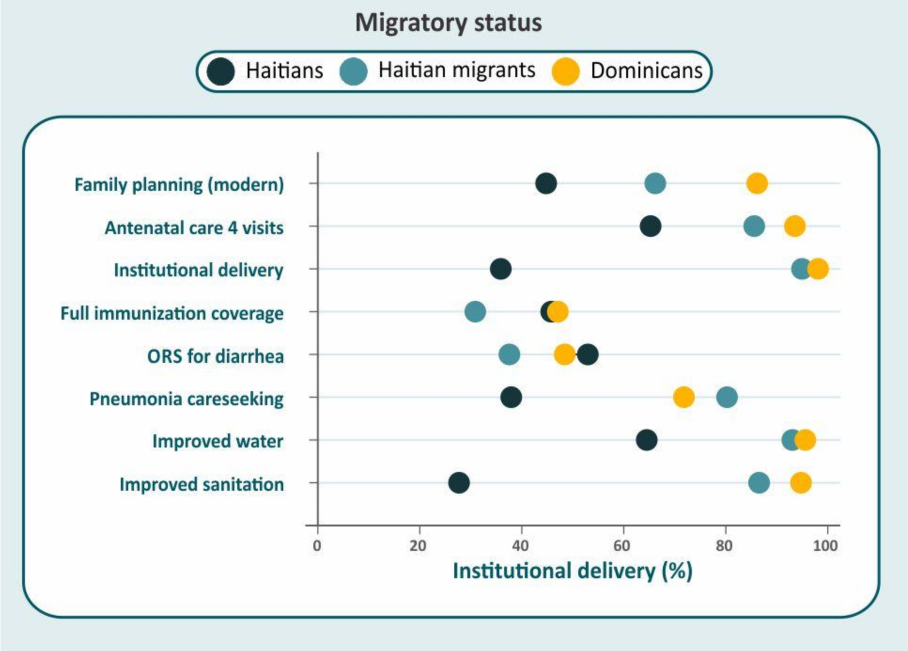

Figure A1. Comparison of coverage with selected RMNCH interventions in Haitians living in Haiti (2012 DHS), Haitian migrants living in the Dominican Republic and native Dominicans (2014 MICS).

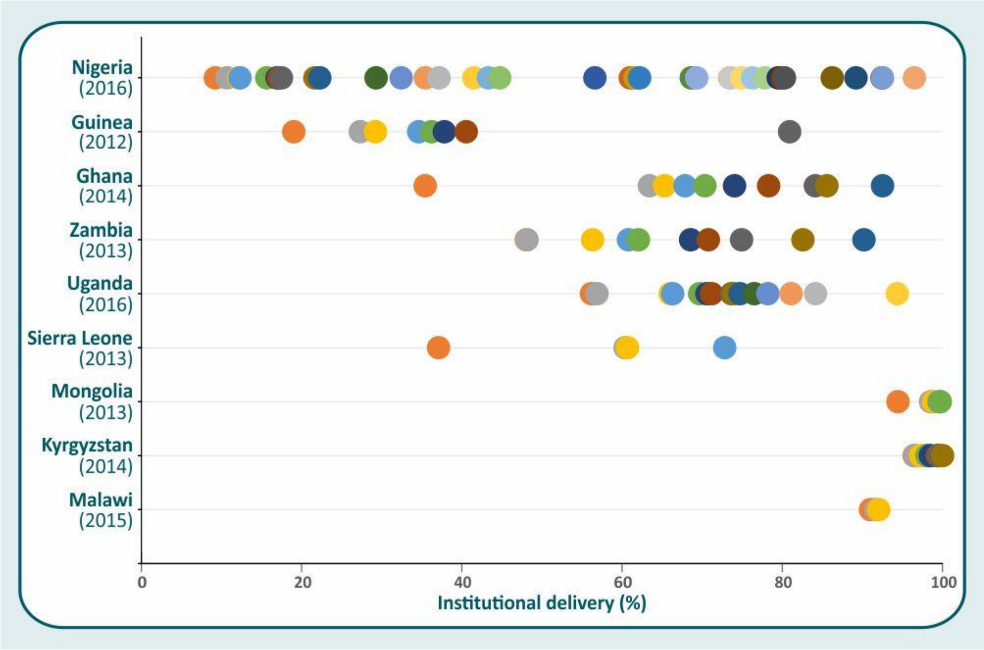

Figure A2. Institutional delivery coverage by subnational region in selected countries with varied patterns of geographic inequalities. Each circle represents one geographic domain in the national survey.

All else being equal, geographic inequalities will be wider in countries with many subnational domains, compared to those with few domains. Statistical methods are available, however, that allow the comparison of the magnitude of geographic inequalities in countries with different

numbers of domains.[1] Figure A2 shows examples of countries with wide and with narrow geographic disparities; the latter tend to show higher national coverage levels than the former.

There have been important advances in using information from surveyed clusters in a country to produce geostatistically modelled surfaces, often with 5 km resolution, for mortality, nutrition and coverage outcomes.[2] (https://spatialdata.dhsprogram.com/modeled-surfaces/). Analyses of geographic disparities will also benefit from a combination of administrative data from health management information systems and survey results that can validate the quality of routine information. [3]

Age

Growing interest in adolescent health in general,[4] and particularly in girl marriage and childbearing,(IAP 2016) led Countdown to 2015 to incorporate age-stratified analyses in 2014 for reproductive and child health indicators.[5] Age of the woman at the time of the survey is used for reproductive and child health coverage indicators, whereas age at childbirth is used for maternal health indicators including antenatal, delivery and post-partum care.

Information on age is straightforward to collect, yet analyses of young adolescent girls face two restrictions even for reproductive and child health where surveys more commonly include adolescents. First, only two surveys (Bangladesh and Colombia) in our database carried out since 2010 include information on 10-14-year-old girls. Second, the number of adolescents, particularly of 15-17-year-olds is often too small for RMNCH analyses. The median number of women in this group, in surveys carried out since 2010, is only 69, whereas the median number for 18-19-year-olds is 216. This is because most surveys only collect information on women who report being married or in a union, or sexually active. In some societies, openly asking about sexual activity for unmarried adolescents is sensitive which results in many surveys failing to ask such questions.

Figure A3 shows that institutional delivery coverage according to maternal age presents different patterns in different countries. Whereas young adolescent mothers show lower coverage in some countries, in others they show higher coverage than older women.

Disability

Of 129 surveys carried out since 2010, only 22 included some information on disability. Most included a limited number of questions on vision, hearing, movement and mental disability, with a few exceptions such as Mexico where the MICS included an extensive module on the topic. The wording of the questions varies from survey to survey, which limits cross-country comparisons. Sample sizes will also make it difficult to stratify health and coverage outcomes according to the presence of disability.

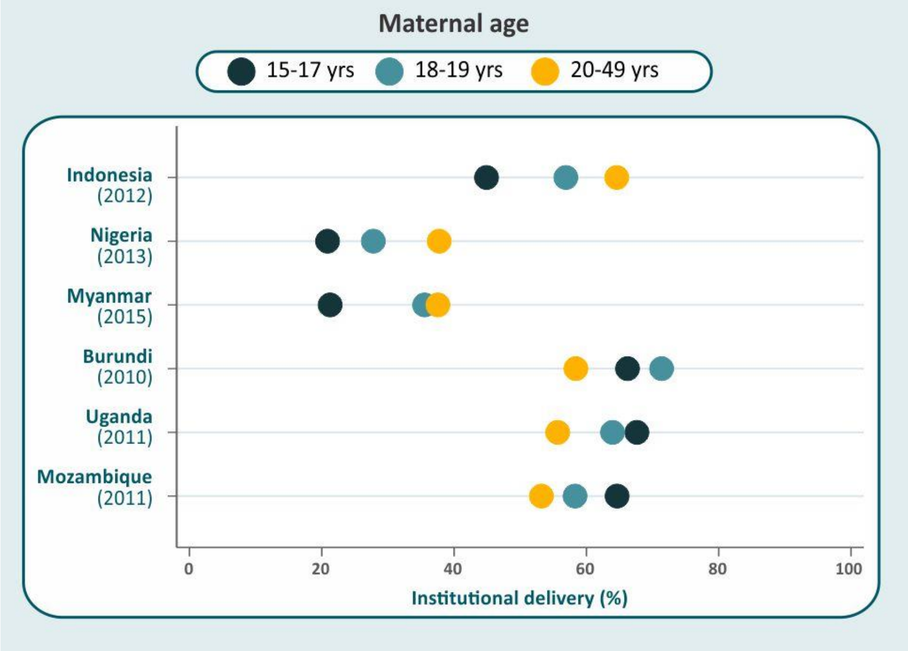

Figure A3. Institutional delivery coverage according to age of the woman in selected countries.

Education

Despite education or schooling of the woman (or mother for child indicators) being probably the most commonly used socioeconomic stratifier in RMNCH prior to the advent of asset indices [6], yet it is not mentioned in SDG 17.18. Years of schooling are relatively easy to collect, although changes in school curricula over time need to be accounted for. Schooling can also be incorporated in analyses of intersectionality, for example with urban/rural residence or sex of the child.

Possibly the greatest drawback of education is heaping, for example with substantial proportions of the women with less than complete primary education in low-income countries, in contrast with large numbers of women with secondary or higher education in upper-middle income countries.

Because the relative sizes of the educational categories vary by country as well as over time in the same countries, simple comparisons between the extreme educational categories – e.g. none versus secondary or higher – should be interpreted with caution. An alternative is to use summary indices of inequality such as the slope or concentration indices.[7] These indices are derived from the full distribution of the health outcome in all educational categories, and account for differences in the sizes of these categories in each survey.

References

1. Hosseinpoor AR, Bergen N, Barros AJ, Wong KL, Boerma T, Victora CG. Monitoring subnational regional inequalities in health: measurement approaches and challenges. *Int J Equity Health* 2016; **15**(1): 18. doi: 10.1186/s12939-016-0307-y.

2. Golding N, Burstein R, Longbottom J, et al. Mapping under-5 and neonatal mortality in Africa, 2000-15: a baseline analysis for the Sustainable Development Goals. *Lancet* 2017; **390**(10108): 2171-82. doi: 10.1016/s0140-6736(17)31758-0.

3. Hosseinpoor AR, Bergen N. Area-based units of analysis for strengthening health inequality monitoring. *Bull World Health Organ* 2016; **94**(11): 856-8. doi: 10.2471/blt.15.165266.

4. Patton GC, Sawyer SM, Santelli JS, et al. Our future: a Lancet commission on adolescent health and wellbeing. *Lancet* 2016; **387**(10036): 2423-78. doi: 10.1016/s0140-6736(16)00579-1.

5. Countdown to 2015. Countdown to 2015: fulfilling the health agenda for women and children. The 2014 report. Geneva: WHO; 2014.

6. Cleland J, Bicego G, Fegan G. Socioeconomic inequalities in childhood mortality: the 1970s to the 1980s. *Health Transit Rev* 1992; **2**(1): 1-18.

7. Barros AJ, Victora CG. Measuring Coverage in MNCH: Determining and Interpreting Inequalities in Coverage of Maternal, Newborn, and Child Health Interventions. *PLoS Med* 2013; **10**(5): e1001390. doi: 10.1371/journal.pmed.1001390.
